# MiR-340 Is a Biomarker for Selecting Treatment Between Chemotherapy and Allogeneic Transplantation in Acute Myeloid Leukemia

**DOI:** 10.3389/fonc.2019.01058

**Published:** 2019-10-11

**Authors:** Mingshan Niu, Ninghan Zhang, Rong Wang, Tingting Shao, Yuan Feng, Yangling Shen, Xuejiao Liu, Kai Zhao, Shengyun Zhu, Linyan Xu, Yao Yao, Kailin Xu

**Affiliations:** ^1^Blood Diseases Institute, Affiliated Hospital of Xuzhou Medical University, Xuzhou Medical University, Xuzhou, China; ^2^Department of Hematology, Affiliated Hospital of Xuzhou Medical University, Xuzhou, China; ^3^Department of Hematology, The Third Affiliated Hospital of Soochow University, Changzhou, China; ^4^Department of Neurosurgery, Institute of Nervous System Diseases, Xuzhou Medical University, Xuzhou, China

**Keywords:** miR-340, acute myeloid leukemia, clinical outcome, chemotherapy, allo-HCST

## Abstract

Acute myeloid leukemia (AML) requires refined risk stratification tools to drive decisions concerning effective therapeutic strategies. Here, genome-wide screening was carried out for identifying miRNA molecules capable of predicting treatment outcome in AML patients based on the TCGA dataset. We identified miR-340 as a prognostic factor for selecting treatment between chemotherapy and allogeneic transplantation (allo-HSCT). In multivariable analyses, low miR-340 expression independently predicted reduced OS (HR = 2.07, *P* = 0.004) and EFS (HR = 1.909, *P* = 0.01) independent of other well-known prognostic factors. Meanwhile, allo-HSCT overcome deleterious outcomes related to low miR-340. Cases administered allo-HSCT showed markedly improved OS (HR = 0.316, *P* < 0.0001) and EFS (HR = 0.391, *P* = 0.002) in comparison with those receiving chemotherapy in the low miR-340 group. Gene expression assessment revealed that elevated miR-340 amounts were negatively correlated with HOXA/HOXB cluster levels, as well as the amounts of the HOX cofactor MEIS1. Strikingly, *in silico* analysis pointing to HOXA10, HOXB2, and MEIS1 as miR-340 targets. The miR-340 expression may help identify cases requiring strategies for selecting the optimal therapeutic option between chemotherapy and allo-HCST. AML cases showing low miR-340 levels should be strongly considered for early allo-HSCT treatment.

## Introduction

Acute myeloid leukemia (AML), the commonest acute leukemia type affecting adult individuals, results from a fast clonal expansion of cancerous myeloblasts (1, 2). AML has a complex and heterogeneous nature (3). Thus, it shows variable outcomes in affected individuals upon administration of current treatments. Cytotoxic chemotherapeutics are considered the mainstay therapeutic option for AML (4, 5). Meanwhile, allogeneic hematopoietic stem cell transplantation (allo-HSCT) provides potent antileukemic effects and potential cure in high-risk patients (6, 7). In AML cases, prognosis is associated with multiple intrinsic parameters such as cytogenetic and genetic modifications. For example, NPM1, CEBPA, FLT3, IDH1, IDH2, and TET2 mutations show associations with patient outcome, and are used for AML prognosis (8–10). Although leukemogenesis is mechanistically well-understood, most AML cases remain uncured. Indeed, no currently available classification system is completely accurate. This suggests an urgent need for identifying new prognostic markers to further ameliorate AML stratification and select rational treatment options.

MicroRNAs (miRNAs) specifically bind mRNA targets and reduce the expression of encoded proteins by translation inhibition (11). Dysregulated miRNAs in AML affects cell growth and hematopoietic differentiation (12, 13). Furthermore, aberrant expression of miRNAs is related to patient outcome (14). For instance, elevated miR-181a content predicts favorable outcome in AML with normal cytogenetic features (15). Patients expressing elevated miR-212 amounts tend to show longer survival in all cytogenetic subtypes (16). Meanwhile, overexpression of miR-3151 is related to decreased overall and disease-free survival in cytogenetically normal AML (17). Moreover, combination of miR-3151 with the associated host gene BAALC is more efficient in predicting worse outcome. However, most published reports did not distinguish the effects of chemotherapeutics and allo-HSCT on treatment outcome. It is generally accepted that a marker's value in AML prognosis depends on the therapeutic method administered. Thus, miRNAs could show distinct prognostic values between chemotherapy and allo-HSCT treatment.

Here, we performed genome-wide screening to identify miRNAs with significant prognostic values in AML cases administered chemotherapeutics. We identified miR-340 as a prognostic factor independently of reported clinico-molecular predictive factors in AML. In addition, whether all-HCST overcomes the adverse prognostic impact of miR-340 expression was assessed. To assess molecular mechanisms, the expression levels of genes and miRNAs throughout the genome were determined.

## Methods

### Patients

All the 162 cases assessed were newly diagnosed AML (non-M3 FAB subtypes) based on the WHO classification. RNA-Seq data for the above AML cases were provided by The Cancer Genome Atlas (Project ID: TCGA-LAML) (18). These AML cases were included in a single-institution tissue banking protocol that had approval from the human studies committee at Washington University (WU HSC #01-1014). All samples were collected from patients with previously untreated *de novo* AML. MicroRNA and mRNA levels were determined before treatment. The risk classification of AML is based on the NCCN recommendation which includes cytogenetics as well as gene mutational status. Patient treatment followed the NCCN guidelines. Among the 162 cases, 148 (91.4%) received standard induction chemotherapy (3 and 7 days of anthracycline and cytarabine, respectively); the remaining 14 patients received decitabine or azacitidine. Patients with unfavorable risk underwent allo-HSCT if medically suitable for transplantation and there was a matched donor. Some cases with intermediate risk were also administered allo-HSCT during the disease course. A total of 90 cases were solely administered chemotherapy, while 72 also underwent allo-HSCT. Clinical data, such as treatment approach and outcomes, can be found on the TCGA website (Supplementary Table 1).

### Gene-Expression Profiling

One hundred and fifty-five of the 162 cases were assessed for both miRNA and mRNA expression levels. Their specimens were employed to identify genes associated with miR-340 expression. In mRNA-seq, genes showing expression levels equal or inferior to a noise threshold of RPKM (Reads per kilobase per million mapped reads) in ≥75% of specimens were excluded. In miRNA-seq, RPM (Reads per million reads) was employed for normalization. Data underwent log2 transformation pre-analysis. The mRNA levels were compared between the high and low miRNA groups, with a univariate significance level at 0.005 to identify differentially expressed genes (DEGs). Then, hierarchical clustering using the multiple experiment viewer (MeV) 4.9.0 software was carried out to order gene rows; miRBase Targets V7 and Targetscan Release 7.1 were used for predicting microRNA targets. Gene Ontology enrichment was performed of genes associated with miR-340 using the Database for Annotation, Visualization, and Integrated Discovery (DAVID).

### Statistical Analysis

Baseline features were compared between the high and low miRNA groups. Median levels of miR-340 were employed to identify patients with low and high miRNA levels. The miR-340 median values in the chemotherapy and allo-HSCT groups were 82.1 (13.2–227.5) and 75.0 (18.9–286.1), respectively. The Mann-Whitney *U*-test was employed to assess associations of two continuous variables. Fisher's exact test or the chi-square test was employed for categorical variables. Overall survival (OS) was defined as time between case enrolment and death, censoring live cases at final follow-up. Event-free survival (EFS) was defined as time between case enrolment and induction failure, recurrence, or death. Estimated OS and EFS distributions were determined by Kaplan-Meier curves, with the log-rank test carried out for comparing survival data.

The univariate Cox proportional hazards model was employed for evaluating associations of 340 expression with OS and EFS. The multivariate Cox proportional hazards model was used for identifying factors that affect survival. The factors assessed for model inclusion comprised miR-340 expression amounts; FLT3-ITD, NPM1, DNMT3A, RUNX1, TP53, TET2, MLL-PTD, IDH1/IDH2, and NRAS/KRAS mutation statuses; age; karyotype; and WBC involvement. Parameters with *p* = 0.10 in univariate analysis were further assessed by multivariate analysis (backward selection), in which *p* < 0.05 indicated significance. R v3.1.5, GraphPad Prism and SPSS were employed for data analysis, and *P* < 0.05 indicated statistically significant differences.

## Results

### Associations of miR-340 Levels With Clinico-Molecular Features

The chemotherapy and allo-HSCT groups were each subdivided into two according to median amounts of miRNAs, respectively. Tables 1, 2 summarize the associations of clinico-genetic features with miR-340 levels. In the chemotherapy group, cases with low miR-340 levels showed a higher rate of first relapse or death within 1 year after enrolment in comparison with those expressing high miR-340 amounts (82.2 vs. 55.6%, *P* = 0.012). There were no significant associations of other miR-340 expression distributions with clinical features. The above findings suggested that miR-340 expression may predict prognosis independently from known molecular features.

**Table 1:** Comparison of clinical characteristics with miR-340 expression in AML patients.

**Characteristic**	**Chemotherapy group**			**Allo-HSCT group**		
	**High miR-340 (*n* = 45)**	**Low miR-340 (*n* = 45)**	***P***	**High miR-340 (*n* = 36)**	**Low miR-340 (*n* = 36)**	***P***
						
Age/years, median	66 (22–81)	66 (31–88)	0.913	45.5 (21–72)	55.5 (18–65)	0.022
Age group/*n* (%)			0.652			0.188
<60 years	16 (35.6)	13 (28.9)		29 (80.6)	23 (63.9)	
≥60 years	29 (64.4)	32 (71.1)		7 (19.4)	13 (36.1)	
Gender/*n* (%)			0.137			1.000
Male	29 (64.4)	21 (46.7)		21 (55.6)	21 (58.3)	
Female	16 (35.6)	24 (53.3)		16 (44.4)	15 (41.7)	
WBC/ ×10^9^/L, median	16 (1.5–298.4)	15.2 (0.7–297.4)	0.840	31.6 (1.2–223.8)	23.6 (0.6–118.8)	0.209
BM blast/%, median	67 (30–98)	76 (32–99)	0.056	70.5 (34–91)	70 (30–100)	0.191
PB blast/%, median	37 (0–97)	23 (0–98)	0.929	53 (0–96)	37 (0–91)	0.054
FAB subtypes/*n* (%)						
M0	3 (6.7)	5 (11.1)	0.549	3 (8.3)	6 (16.7)	0.478
M1	9 (20.0)	11 (24.4)	0.800	15 (41.7)	8 (22.2)	0.129
M2	14 (31.1)	7 (15.6)	0.134	12 (33.3)	7 (19.4)	0.285
M4	13 (28.9)	11 (24.4)	0.812	5 (13.9)	9 (25.0)	0.372
M5	4 (8.9)	9 (20)	0.230	0	4 (11.1)	0.115
M6	0 (0)	1 (2.2)	1.000	0	1 (2.8)	1.000
M7	1 (2.2)	1 (2.2)	1.000	0	1 (2.8)	1.000
Others	1 (2.2)	0 (0)	1.000	1 (2.2)	0	1.000
Karyotype/*n* (%)						
Normal	21 (46.7)	23 (51.1)	0.833	13 (36.1)	21 (58.3)	0.098
Complex	6 (13.3)	6 (13.3)	1.000	6 (16.7)	6 (16.7)	1.000
8 Trisomy	2 (4.4)	0 (0)	0.494	0	4 (11.1)	0.115
CBFβ-MYH11	5 (11.1)	2 (4.4)	0.434	5 (13.9)	0	0.054
11q23/MLL	1 (2.2)	3 (6.7)	0.616	0	3 (8.3)	0.239
−7/7q-	1 (2.2)	2 (4.4)	1.000	3 (8.3)	0	0.239
BCR-ABL1	1 (2.2)	0	1.000	2 (5.6)	0	0.493
RUNX1-RUNX1T	5 (11.1)	1 (2.2)	0.203	1 (2.8)	0	1.000
Others	3 (6.7)	8 (17.8)	0.197	6 (16.7)	2 (5.6)	0.260
Risk/*n* (%)^a^						
Good	10 (22.2)	3 (6.7)	0.069	6 (16.7)	0	0.025
Intermediate	24 (53.3)	26 (57.8)	0.832	15 (41.7)	26 (72.2)	0.017
Poor	10(22.2)	15 (33.3)	0.233	14 (38.9)	10 (37.8)	0.454
Others	1 (2.2)	1 (2.2)	1.000	1 (2.8)	0(0)	1.000
Induction regimen			1.000			1.000
7 + 3	38	38		35	36	
Decitabine	6	7		1	0	
Azacitidine	1	0		0	0	
Donor types						0.021
MUD	–	–		23(63.9)	15(41.7)	
Sibling	–	–		11(30.6)	21(58.3)	
Haploidentical	–	–		2(5.5)	0(0)	
Relapse/*n* (%)^b^			0.012			0.48
Yes	25 (55.6)	37 (82.2)		16 (44.4)	20 (55.6)	
No	20 (44.4)	8 (17.8)		20 (55.6)	16 (44.4)	

Mann-Whitney test was used for continuous variables. Chi-square tests were used for categorical variables.

WBC, white blood cell; BM, bone marrow; PB, peripheral blood; FAB, French-American-British classification; MUD, Matched unrelated donor.

a The risk classification of AML is based on NCCN recommendation which includes cytogenetics as well as gene mutational status.

b *First Relapse or death within one year*.

**Table 2:** Comparison of molecular characteristics with miR-340 expression in AML patients.

**Characteristic**	**Chemotherapy group**			**Allo-HSCT group**		
	**High miR-340 (*n* = 45)**	**Low miR-340 (*n* = 45)**	***P***	**High miR-340 (*n* = 36)**	**Low miR-340 (*n* = 36)**	***P***
						
FLT3-ITD/*n* (%)			0.409			0.155
Presence	6 (13.3)	10 (22.2)		5 (13.9)	11 (30.6)	
Absence	39 (86.7)	35 (77.8)		31 (86.1)	25 (69.4)	
NPM1/*n* (%)			0.175			0.003
Mutation	11 (24.4)	18 (40.0)		4 (11.1)	16 (44.4)	
Wild type	34 (75.6)	27 (60.0)		32 (88.9)	20 (55.6)	
CEBPA/*n* (%)						
Single mutation	1 (2.2)	2 (4.4)	1.000	4 (11.1)	1 (2.8)	0.357
Double mutation	0	0		3 (8.3)	0	0.239
Wild type	44 (97.8)	43 (95.6)	1.000	29 (80.6)	35 (97.2)	0.055
DNMT3A/*n* (%)			0.157			0.055
Mutation	9 (20.0)	16 (35.6)		5 (13.9)	13 (36.1)	
Wild type	36 (80.0)	29 (64.4)		31 (86.1)	23 (63.9)	
IDH1/IDH2/*n* (%)			0.409			1.000
Mutation	10 (22.2)	6 (13.3)		9 (25.0)	9 (25.0)	1.000
Wild type	35 (77.8)	41 (86.7)		27 (75.0)	27 (75.0)	
RUNX1/*n* (%)			0.714			0.710
Mutation	5 (1.1)	3 (6.7)		3 (8.3)	5 (13.9)	
Wild type	40 (88.9)	42 (93.3)		33 (91.7)	31 (86.1)	
MLL-PTD/*n* (%)			1.000			0.614
Presence	3 (6.7)	2 (4.4)		1 (2.8)	3 (8.3)	
Absence	42 (93.3)	43 (95.6)		35 (97.2)	33 (91.7)	
NRAS/KRAS/*n* (%)			1.000			0.429
Mutation	7 (15.6)	6 (13.3)		5 (11.1)	2 (2.8)	
Wild type	38 (84.4)	39 (86.7)		31 (88.9)	34 (97.2)	
TET2/*n* (%)			0.758			1.000
Mutation	7 (15.6)	5 (11.1)		2 (5.6)	2 (5.6)	
Wild type	38 (84.4)	40 (88.9)		33 (94.4)	34 (94.4)	
TP53/*n* (%)			0.522			0.614
Mutation	7 (15.6)	4 (8.9)		1 (2.8)	3 (8.3)	
Wild type	38 (84.4)	41 (91.1)		35 (97.2)	33 (91.7)	

*Chi-square tests were used for categorical variables*.

### Prognostic Value of miR-340 in Cases Administered Chemotherapy or Allo-HSCT

**Figure 1:**
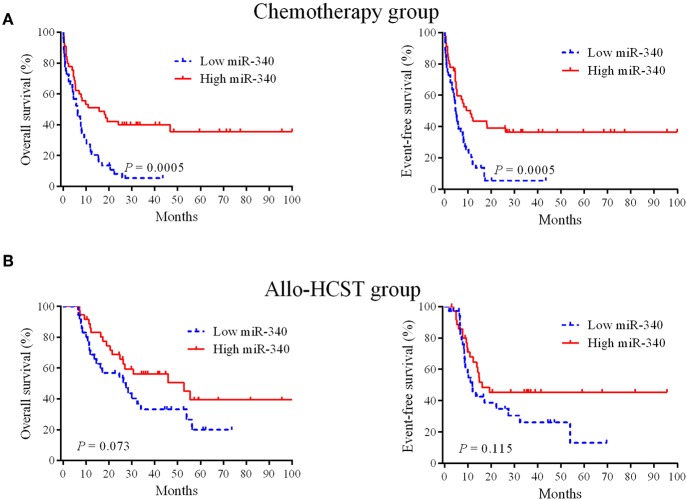
Kaplan-Meier survival curves based on miR-340 expression. **(A)** Cases highly expressing miR-340 showed markedly prolonged OS and EFS in the chemotherapy group (*n* = 90). **(B)** Effects of miR-340 levels on OS and EFS in cases administered allo-HSCT (*n* = 72).

To obtain prognostic markers for refining AML stratification, a genome-wide screen of miRNAs in AML patients was carried out; miR-340 was detected as a novel molecule predicting prognosis in AML cases treated by chemotherapy. In the chemotherapy group, low miR-340 expressers showed starkly reduced OS (*P* = 0.0005) and EFS (*P* = 0.0005) in comparison with high expressers (Figure 1A). Notably, 5 year OS rates were 35.6 and 5.4% in the high and low miR-340 expression groups, respectively. However, miR-340 levels were not associated with patient outcome in AML after allo-HCST (Figure 1B). The above findings indicated that low miR-340 was an unfavorable prognostic factor in AML cases administered chemotherapy.

### MiR-340 Is Independently Associated With Clinical Outcome in Patients With AML

To assess whether miR-340 levels independently predict survival in AML, univariable and multivariate cox analyses were carried out. Univariate analysis (Table 3) revealed low miR-340 had a significant association with reduced OS (HR = 2.393, *P* = 0.001) and EFS (HR = 2.383, *P* = 0.001) in patients treated by chemotherapy. In multivariate analysis, miR-340 and many known prognostic factors were evaluated (Table 3). Low miR-340 expression was independently predictive of reduced OS (HR = 2.07, *P* = 0.004) and EFS (HR = 1.909, *P* = 0.01) upon adjustment for age (*P* < 0.0001) and karyotype risk (*P* < 0.0001). In patients administered allo-HSCT, univariate analysis revealed TP53-mutant cases displayed adverse OS (*P* = 0.009); however, the miR-340 expression status was not associated with OS and EFS (Table 4). Multivariable analysis showed TP53 and FLT3-ITD mutations were independently predictive of reduced OS (*P* = 0.003 and *P* = 0.031, respectively). Therefore, the miR-340 expression status did not remain a survival predictor after allo-HSCT.

**Table 3:** Univariate and multivariate analyses in patients treated with chemotherapy.

**Variables**	**EFS**		**OS**	
	**HR (95% CI)**	**P-value**	**HR (95% CI)**	***P*-value**
**Univariate analyses**				
MiR-340 (low vs. high)	2.383 (1.455–3.903)	0.001	2.393 (1.458–3.927)	0.001
Age (≥60 vs. <60)	3.560 (1.960–6.467)	0.000	3.328 (1.838–6.024)	0.000
Karyotype^a^	3.827 (2.181–6.716)	0.000	4.069 (2.309–7.170)	0.000
WBC (≥20 vs. <20 × 10^9^/L)	1.015 (0.633–1.627)	0.952	0.980 (0.611–1.571)	0.932
FLT3-ITD (positive vs. negative)	1.095 (0.587–2.040)	0.776	1.049 (0.563–1.956)	0.880
NPM1 (mutated vs. wild)	1.050 (0.633–1.741)	0.850	0.965 (0.582–1.599)	0.890
DNMT3A (mutated vs. wild)	1.301 (0.774–2.185)	0.320	1.299 (0.775–2.179)	0.321
RUNX1 (mutated vs. wild)	1.502 (0.717–3.147)	0.281	1.591 (0.759–3.335)	0.219
TP53 (mutated vs. wild)	3.011 (1.539–5.892)	0.001	2.898 (1.487–5.649)	0.002
TET2 (mutated vs. wild)	0.778 (0.372–1.625)	0.504	0.686 (0.328–1.434)	0.316
MLL-PTD (mutated vs. wild)	0.891 (0.324–2.445)	0.822	0.945 (0.344–2.596)	0.913
IDH1/IDH2 (mutated vs. wild)	0.973 (0.271–1.273)	0.926	0.988 (0.550–1.777)	0.969
NRAS/KRAS (mutated vs. wild)	1.214 (0.637–2.314)	0.556	1.228 (0.644–2.340)	0.532
**Multivariate analyses**				
MiR-340 (low vs. high)	1.909 (1.164–3.132)	0.010	2.070 (1.260–3.400)	0.004
Age (≥60 vs. <60)	3.555 (1.916–6.598)	0.000	3.103 (1.672–5.758)	0.000
Karyotype^a^	3.925 (2.151–7.161)	0.000	3.762 (2.088–6.779)	0.000
WBC (≥20 vs. <20 × 10^9^/L)	1.670 (0.998–2.794)	0.051	1.678 (0.997–2.822)	0.051
DNMT3 (mutated vs. wild)	1.677 (0.968–2.905)	0.065	–	–

EFS, Event-free survival; OS, Overall survival; WBC, white blood cell.

a *Unfavorable cytogenetics vs. others*.

**Table 4:** Univariate and multivariate analyses in patients treated with allo-HSCT.

**Variables**	**EFS**		**OS**	
	**HR (95% CI)**	***P*-value**	**HR (95% CI)**	***P*-value**
**Univariate analyses**				
MiR-340 (low vs. high)	1.627 (0.881–3.005)	0.120	1.738 (0.941–3.208)	0.077
Age (≥60 vs. <60)	0.873 (0.438–1.741)	0.700	1.190 (0.594–2.382)	0.624
Karyotype^a^	1.241(0.659–2.339)	0.504	1.376 (0.731–2.591)	0.323
WBC (≥20 vs. <20 × 10^9^/L)	1.089 (0.594–1.999)	0.782	0.826 (0.450–1.516)	0.537
Remission status^b^	0.798 (0.381–1.670)	0.549	0.616 (0.293–1.296)	0.202
Donor types^c^	0.941 (0.514–1.725)	0.845	0.713 (0.388–1.310)	0.276
FLT3-ITD (positive vs. negative)	1.876 (0.914–3.851)	0.086	1.973 (0.953–4.084)	0.067
NPM1 (mutated vs. wild)	1.007 (0.515–1.970)	0.983	1.023 (0.523–1.998)	0.948
DNMT3A (mutated vs. wild)	1.285 (0.655–2.520)	0.466	1.387 (0.704–2.731)	0.344
RUNX1 (mutated vs. wild)	1.145 (0.449–2.290)	0.777	1.579 (0.613–4.067)	0.344
TP53 (mutated vs. wild)	2.034 (0.718–5.760)	0.181	4.334 (1.453–12.925)	0.009
TET2 (mutated vs. wild)	0.526 (0.127–2.186)	0.377	0.670 (0.162–2.776)	0.581
MLL-PTD (mutated vs. wild)	5.775 (1.664–20.042)	0.006	2.728 (0.832–8.944)	0.098
IDH1/IDH2 (mutated vs. wild)	0.587 (0.271–1.273)	0.177	0.633 (0.293–1.368)	0.245
NRAS/KRAS (mutated vs. wild)	0.796 (0.245–2.586)	0.705	0.488 (0.150–1.587)	0.233
**Multivariate analyses**				
MLL-PTD (mutated vs. wild)	5.775 (1.664–20.042)	0.006	–	–
FLT3-ITD (positive vs. negative)	–	–	2.274 (1.080–4.788)	0.031
TP53 (mutated vs. wild)	–	–	5.361 (1.746–16.454)	0.003

EFS, Event-free survival; OS, Overall survival; WBC, white blood cell.

a Unfavorable cytogenetics vs. others.

b CR vs. non-CR.

c *Matched unrelated donor (MUD) vs. matched sibling + haploidentical donors*.

### Allo-HSCT Overcomes the Adverse Prognostic Effects of Low miR-340 Expression in AML

**Figure 2:**
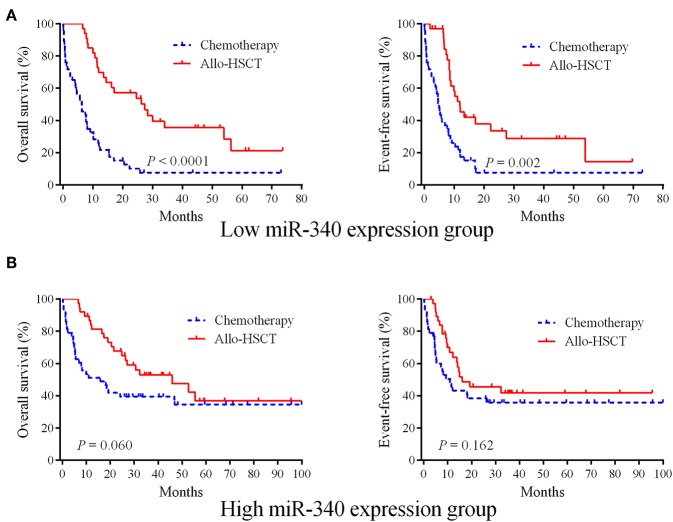
Allo-HSCT overcomes the adverse prognostic influence of low miR-340 expression in AML. **(A)** The 162 cases were divided into two groups according to median miR-340 levels. Kaplan-Meier survival curves for cases administered chemotherapy (*n* = 47) and allo-HSCT (*n* = 34), respectively, in the low miR-340 group. **(B)** Kaplan-Meier survival curves for cases administered chemotherapy (*n* = 43) and allo-HSCT (*n* = 8), respectively, in the high miR-340 group.

Whether allo-HSCT overcomes the unfavorable effects of low miR-340 expression was assessed. The 162 cases were assigned to two groups based on miR-340 levels. Then, each group was further divided into the chemotherapy and allo-HSCT groups. In cases with low miR-340 expression, significantly longer OS (HR = 0.316, 95% CI 0.167–0.459, *P* < 0.0001) and EFS (HR = 0.391, 95% CI 0.231–0.622, *P* = 0.002) were observed after allo-HSCT (Figure 2A). However, there were no differences in OS (*P* = 0.060) and EFS (*P* = 0.162) between the allo-HSCT and chemotherapy groups in high miR-340 expressers (Figure 2B). The above findings indicated miR-340 might constitute a novel molecule for detecting cases requiring strategies to select a rational therapeutic management.

### Biological Insights Into miR-340 Function in AML

**Figure 3:**
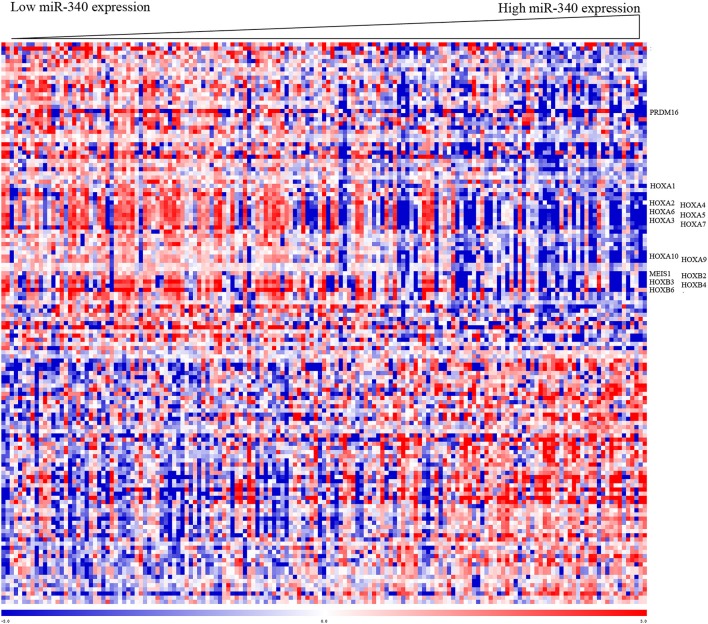
Heat map of the gene expression signature related to miR-340 expression in AML. Cases (columns) were ordered from left to right by increasing miR-340 levels. Genes (rows) were ordered by hierarchical cluster analysis. Blue and red reflect expression levels lower and above median values for the indicated genes, respectively; miR-340 associated genes are indicated.

To gain insights into the biological functions of miR-340, gene expression signatures related to the miRNA expression were assessed in AML patients. As shown in Figure 3, the expression levels of 135 genes showed significant associations with miR-340 expression, with 61 and 74 positively and negatively correlated, respectively. In this study, miR-340 expression was negatively associated with HOXA/HOXB cluster and MEIS1 amounts. The latter genes are important in AML leukemogenesis and self-renewal features in leukemic stem cells (19). We further analyzed the biological significance of these genes by separating AML cases into two groups according to median miR-340 expression. In the low miR-340 expression group, the HOXA1 (*P* = 0.0112), HOXA2 (*P* = 0.019), HOXA3 (*P* < 0.0001), HOXA4 (*P* = 0.0005), HOXA5 (*P* < 0.0001), HOXA6 (*P* < 0.0001), HOXA7 (*P* < 0.0001), HOXA9 (*P* = 0.0004), HOXA10 (*P* < 0.0001), HOXB2 (*P* = 0.0026), HOXB3 (*P* < 0.0001), HOXB4 (*P* < 0.0001), HOXB6 (*P* < 0.0001), MEIS1 (*P* = 0.0001), and PRDM16 (*P* = 0.0002) genes were all expressed at higher levels compared with high miR-340 expression group. Notably, the histone H3K4 methyltransferase PRDM16, a gene that predicts adverse outcome in AML (20), was downregulated in the high miR-340 group. Strikingly, HOXA10, HOXB2, MEIS1 and PRDM16 were identified as miR-340 targets by *in silico* prediction. These findings corroborated clinical data for AML generated by miRNA analysis.

Gene Ontology revealed that genes contributing to biological regulation, cellular and developmental processes, transcription regulation, immune system process, cell apoptosis and proliferation, myeloid cell differentiation, hematopoietic organ development, cellular response to chemical stimulus and embryonic morphogenesis were remarkably overrepresented among DEGs related to miR-340 expression (Table 5).

**Table 5:** Gene ontology terms of biological processes in the miR-340 associated expression profile.

**GO ID**	**GO Terms**	**Percentage of members of the GO term present in the miR-363 profile**	***P*-value**
GO:0065007	Biological regulation	71.8	0.001
GO:0050794	Regulation of cellular process	65.3	0.004
GO:0032501	Multicellular organismal process	55.6	<0.001
GO:0032502	Developmental process	46.0	<0.001
GO:0048731	System development	38.7	<0.001
GO:0006355	Regulation of transcription, DNA-templated	24.2	0.007
GO:0070887	Cellular response to chemical stimulus	20.2	0.043
GO:0002376	Immune system process	19.4	0.036
GO:0042981	Regulation of apoptotic process	13.7	0.015
GO:0042127	Regulation of cell proliferation	13.7	0.036
GO:0048598	Embryonic morphogenesis	12.9	<0.001
GO:0009790	Embryo development	12.9	0.001
GO:0048534	Hematopoietic or lymphoid organ development	9.7	0.011
GO:0002520	Immune system development	9.7	0.015
GO:0045637	Regulation of myeloid cell differentiation	4.8	0.007
GO:0045638	Negative regulation of myeloid cell differentiation	4.0	0.029

*GO, Gene Ontology*.

## Discussion

Identifying new prognostic markers is of great importance in adult AML, because currently available molecular stratification approaches do not completely capture the heterogeneity of such cases (21–23). The significance of miRNAs as predictive biomarkers in AML patients remains mostly unclear. The present work demonstrated that miR-340 independently predicted prognosis of AML patients administered chemotherapy, providing an important tool for risk-stratifying AML cases. Of greater importance, allo-HSCT overcame miR-340-associated unfavorable outcomes.

Aberrant miRNA expression has a potential prognostic value in AML (24). High amounts of miR-155 (25), miR-196b (26), miR-362 (27), miR-363 (22), and miR-551b (28) show associations with adverse clinical outcomes. Conversely, elevated amounts of miR-9^*^ (29), miR-34a (30), miR-181a (15), miR-193b (31), miR-212 (16) and miR-425 (14) predict favorable prognosis. As shown above, miR-340 expression independently predicted prognosis in a heterogeneous population of AML cases treated by chemotherapy. Low miR-340 amounts showed associations with reduced survival and risk of disease relapse or death. These findings suggest that miR-340 could enhance the prognostic value of currently known biomarkers in AML. Meanwhile, identifying low miR-340 expression as an adverse prognostic factor could guide intervention with synthetic miR-340 molecules. These findings indicate miR-340 functions independently to impact treatment outcome, and may drive leukemogenesis.

Routine therapeutic approaches after AML remission include conventional chemotherapy and allo-HCST (32). However, prognostic biomarkers efficiently guiding treatment selection are lacking. As demonstrated above, low miR-340 expressers treated by allo-HSCT had remarkably prolonged OS and EFS compared with the chemotherapy group. Notably, no benefit of allo-HSCT was found in cases highly expressing miR-340. The above results suggest that allo-HSCT as first line treatment might be of limited value in cases highly expressing miR-340. Thus, miR-340 levels might help identify individuals requiring strategies for selecting the most suitable therapeutic option between chemotherapy and allo-HCST. Low miR-340 expressing cases may be strongly considered for early allo-HSCT.

The tumor suppressor activity of miR-340 has been described in solid malignancies such as breast, colorectal and non-small cell lung cancers, as well as osteosarcoma and glioblastoma (33–35). However, miR-340's biological role in AML cells is largely undefined. As shown above, miR-340 levels were negatively correlated with HOXA/HOXB cluster and MEIS1 amounts. HOX genes are essential regulators of hematopoietic development and stem cell self-renewal (36). Abnormal overexpression of HOXA and MEIS1 represents a known hallmark of AML (19). Furthermore, artificial overexpression of HOXA7, HOXA9, or HOXA10 in combination with MEIS1 leads to rapid onset AML in animal models (37). High expression of HOXA9 in leukemia blasts predicts adverse outcome in AML. High expression levels of HOXA10 (38, 39) and MEIS1 (40) genes also predict poor outcome in AML. Notably, HOXA10, HOXB2, and MEIS1 were predicted *in silico* to be miR-340 targets in this study. Although they are potential therapeutic targets, developing chemical inhibitors of transcription factor remains challenging. Targeting these genes with synthetic miR-340 compounds may be a potential therapeutic strategy. Taken together, the miR-340 associated gene signature may confirm the notion that AML features miRNA expression changes. However, the mechanisms involved in miR-340 modulation and associated effects on patient outcome in AML after treatment deserve further attention.

## Conclusions

MiR-340 amounts are independently associated with treatment outcome in a highly heterogeneous population of AML patients. MiR-340 amount determination may provide a powerful tool for the identification of a patient subset with adverse outcome in AML, and might help ameliorate stratification risk and inform therapeutic decision making for AML patients. Interestingly, allo-HSCT may overcome miR-340-associated deleterious effects in AML. Therefore, miR-340 expression analysis could help identify patients requiring complex approaches in selecting the most suitable therapeutic option between chemotherapy and allo-HCST.

## Data Availability Statement

All datasets supporting the conclusions contained in the present report are included in the manuscript. All clinical data, including treatment approaches and outcomes, are publicly accessible from the TCGA website (https://cancergenome.nih.gov).

## Author Contributions

MN, NZ, and RW carried out study design and computational analyses. TS, YF, YS, XL, KZ, SZ, and LX participated in statistical analysis. YY and KX contributed to study design and drafted the manuscript. All authors read and approved the final manuscript.

### Conflict of Interest

The authors declare that the research was conducted in the absence of any commercial or financial relationships that could be construed as a potential conflict of interest.

## Supplementary Material

Supplementary Table 1 Clinical data.

## Abbreviations

AML: acute myeloid leukemia
Allo-HSCT: allogeneic hematopoietic stem cell transplantation
TCGA: The Cancer Genome Atlas
OS: overall survival
EFS: event-free survival.
